# Application and prospects of veterinary diagnostic techniques in laboratory monkeys

**DOI:** 10.3389/fvets.2026.1911594

**Published:** 2026-09-29

**Authors:** Zhengjia Zhang, Diangang Han, Qiujiang Wei, Xuewei Fu, Yongqing Yan, Dandan Li, Jige Xin

**Affiliations:** 1 Yunnan Key Laboratory of Veterinary Etiological Biology, College of Veterinary Medicine, Yunnan Agricultural University, Kunming, China; 2 Technology Center of Kunming Customs, Kunming, China; 3 Kunming Yaling Biotechnology Co., Ltd, Kunming, China; 4 Yunnan Dasheng Biotechnology Co., Ltd, Yuxi, China; 5 Animal and Plant Quarantine Center of Haikou Customs, Haikou, China

**Keywords:** disease diagnosis, immunological diagnostic techniques, laboratory monkeys, molecular diagnostic techniques, veterinary diagnostic techniques

## Abstract

As the non-human primate models with the closest genetic relationship to humans, laboratory monkeys are indispensable in life sciences research, drug development, and biological product evaluation. However, infectious diseases either endemic in laboratory monkey colonies or acquired during breeding and husbandry—for example, B virus and *Mycobacterium tuberculosis*-threaten animal welfare and colony security, while also elevating zoonotic transmission risks and compromising the reliability of research data. The diversity, complexity, and clinically insidious nature of diseases in laboratory monkeys impose stringent demands on veterinary diagnostic capabilities. This review summarizes the current status and recent advances in mainstream veterinary diagnostic techniques applied to laboratory monkey disease surveillance. We analyze the specific applications, strengths, and limitations of molecular, immunological, imaging, and conventional microbiological methods for detecting major viral, bacterial, and parasitic pathogens of laboratory monkeys, covering quantitative PCR (qPCR), CRISPR-based assays, ELISA, IFA/IHC, and other established platforms. By comparing these techniques across sensitivity, specificity, cost-effectiveness, and operational feasibility, we delineate their suitability for distinct use cases: high-precision laboratory testing, large-scale colony screening, and point-of-care clinical diagnosis. Current veterinary diagnostic assays for laboratory monkeys achieve robust analytical performance. In several studies, qPCR delivers 100% sensitivity and 100% specificity for B virus detection, digital PCR (dPCR) attains a limit of detection as low as 45 copies/ml for monkeypox virus; and CRISPR/Cas-based assays have demonstrated 100% sensitivity and 100% specificity in clinical validation cohorts. Building on these advances, we further identify practical bottlenecks in diagnostic deployment and propose targeted countermeasures. Finally, we explore emerging trends and application prospects for point-of-care testing, multiplex pathogen detection, and artificial intelligence-assisted diagnosis, which collectively support the development of accurate, efficient, and intelligent disease diagnostics for laboratory monkeys. This work aims to provide a theoretical and practical reference for clinical practice and scientific research focused on laboratory monkey disease prevention and control.

## 1 Hazards of infectious diseases in laboratory monkeys

Laboratory monkeys, primarily including macaques, cynomolgus monkeys, and rhesus monkeys, are non-human primates (NHPs) that share a high degree of anatomical, physiological, genetic, and immunological similarity with humans. As a result, they have become indispensable key experimental animal resources in fundamental medical research, new drug and vaccine development, and disease model construction. However, the widespread use of laboratory monkeys is also accompanied by serious challenges in disease prevention and control. Laboratory monkey populations carry or are susceptible to a wide variety of pathogens, including highly pathogenic zoonotic agents (1, 2).

B virus is widely prevalent in monkey populations, typically presenting a benign course, yet it poses a significant threat to human health, potentially causing encephalomyelitis with a case fatality rate as high as 40% (3, 4); *Mycobacterium tuberculosis (TB)* is also a common pathogen in laboratory monkeys, particularly in Old World monkeys. Tuberculosis not only causes morbidity and mortality in monkey colonies, leading to substantial economic losses, but also represents a serious zoonotic disease that threatens public health security (5, 6). In recent years, with the frequent occurrence of global public health events, emerging or re-emerging zoonotic viruses such as monkeypox virus have garnered significant attention. Their transmission and surveillance in monkey populations are of critical importance (7, 8). In addition, infections with pathogens such as Simian Immunodeficiency Virus (SIV), simian measles virus, *Toxoplasma gondii*, and *Chlamydia spp*. are also common in laboratory monkey populations. These infections may manifest as overt diseases or remain in latent or subclinical states (9–12). Gastrointestinal parasites have also been documented in macaque populations, with potential zoonotic implications (13).

The presence of infectious diseases in laboratory monkeys directly compromises their animal welfare and health, affecting the stability and breeding of the population. Latent infections with pathogens may serve as uncontrolled confounding variables in experimental studies, impairing the accuracy, reproducibility, and reliability of research outcomes. They may even lead to erroneous judgments regarding the safety and efficacy of drugs or therapies. Therefore, the establishment and application of accurate, rapid, and efficient veterinary diagnostic techniques play a critical role in the early detection, precise identification, and effective surveillance of infectious diseases in laboratory monkeys.

Beyond single-pathogen threats, the epidemiological reality in laboratory monkey colonies is further complicated by frequent co-infections. Studies have shown that co-infection with TB and *Simian Retroviru*s (SRV) is prevalent in macaque colonies-with SRV seroprevalence reaching 19~50% in some breeding colonies and TB infection rates as high as 30~45% in endemic facilities. Such co-infections can profoundly impact disease progression, immune responses, and experimental outcomes (2, 9, 14). Concurrent infections with B virus and SIV have also been reported, with B virus seroprevalence reaching 80~90% in adult macaques. SIV infection rates varying from 5 to 40% depending on geographic origin and housing conditions (3, 15, 16). Furthermore, a single macaque can be asymptomatically infected with multiple pathogens simultaneously. For example, co-infection rates of SIV with measles virus or *Toxoplasma gondii* have been documented at 10~30% in certain colonies (9–12, 17). Such co-infections create a complex network of latent infections that acts as an uncontrolled variable in experimental studies, impairing accuracy and reproducibility, and potentially leading to erroneous judgments regarding the safety and efficacy of drugs or therapies. The high prevalence of co - infections underscores the urgent need for diagnostic techniques capable of high - throughput, multiplex detection (18, 19).

Previous reviews have addressed specific aspects of NHP diagnostics: Ohta focused on pathology (2), Yang et al. on viral detection (1), and Wolf et al. on TB testing (20). However, no previous review has integrated all four major diagnostic modalities within a single framework tailored specifically to laboratory monkeys. This Mini Review fills this gap by providing a comprehensive comparative overview across molecular, immunological, imaging, and traditional microbiological methods, clarifying scenario-specific suitability, identifying practical bottlenecks, and discussing emerging technologies. This work serves as a practical reference for veterinarians and researchers working with NHP models.

The diagnosis of infectious diseases in laboratory monkeys carries significant biosafety and occupational health implications that must be carefully addressed. Specimen collection (e.g., blood, saliva, BALF, tissues) should be performed using appropriate restraint and sedation to minimize stress and injury risk. Specimen handling and transport must follow strict biosafety protocols to prevent aerosol generation, spills, and contamination. Containment measures-including biosafety cabinets, sealed centrifuges, and appropriate facility BSL levels-are essential when processing potentially infectious samples. Personal protective measures (e.g., gloves, gowns, face shields, N95 respirators) should be mandatory for all personnel handling monkey-derived specimens or performing necropsies. Decontamination of surfaces, equipment, and waste is critical to prevent environmental contamination. Occupational exposure management-including post-exposure prophylaxis for B virus, TB screening, and incident reporting systems-should be in place for all facilities housing laboratory monkeys (3). The integration of veterinary diagnostics with occupational-health monitoring ensures that diagnostic results from animals inform human health surveillance, creating a bidirectional safety net.

These biosafety and occupational health considerations firmly embed laboratory-monkey diagnostics within the One Health framework. Robust diagnostic practices protect not only animal welfare by enabling early detection and treatment, but also occupational health by safeguarding facility personnel from zoonotic infections. They contribute to zoonotic disease prevention by identifying infected animals before transmission occurs, and to biosafety and biosecurity by containing pathogens at their source. Furthermore, accurate diagnostics enhance public-health surveillance for emerging zoonotic threats and ensure the reliability of biomedical research by maintaining specific-pathogen-free colonies and preventing undetected infections from confounding experimental data. Thus, veterinary diagnostics for laboratory monkeys represent a critical interface between animal and human health, embodying the core principles of One Health (21).

To clearly demonstrate the novelty of this Mini Review, a structured comparison with previous reviews is warranted. Ohta (2) provided a comprehensive summary of pathologic characteristics of infectious diseases in macaques, with a focus on histopathological findings rather than diagnostic methodologies. Yang et al. (1) updated practices for viral detection in breeding macaques, primarily emphasizing molecular assays for specific viruses without coverage of other pathogen categories or non-molecular techniques. Wolf et al. (20) reviewed noninvasive testing strategies for tuberculosis detection in primates, but did not extend to other bacterial, viral, or parasitic pathogens. In contrast, this Mini Review integrates all four major diagnostic modalities-molecular, immunological, imaging, and traditional microbiological methods-across viral, bacterial, and parasitic diseases, and provides a comparative scenario-based framework and a practical diagnostic algorithm. This integrated approach, tailored specifically to the unique constraints of laboratory monkey diagnostics, distinguishes the present work from previous reviews.

Consistent with the Mini Review format, this article does not include a formal systematic review methodology.

## 2 Current status of diagnostic techniques

### 2.1 Limitations of traditional diagnostic techniques

Pathogen isolation and culture have long been regarded as the definitive reference for the diagnosis of bacterial and viral diseases. They enable the recovery of live pathogens and provide a foundation for subsequent antimicrobial susceptibility testing and vaccine development. In the diagnosis of monkey tuberculosis, the isolation and culture of TB from suspicious specimens serve as the definitive basis for confirmation (22). However, traditional culture methods are time-consuming, labor-intensive, and require high-level biosafety facilities. Some pathogens are difficult to culture *in vitro*, which limits their application in rapid and large-scale screening. Conventional serological methods, such as the hemagglutination inhibition test and neutralization test, although still used in population-based epidemiological surveys, are complex to perform and subjective in interpretation. They are gradually being replaced by novel immunological methods such as ELISA (23).

### 2.2 Breakthroughs in novel diagnostic techniques

Molecular diagnostic methods centered on PCR technology have achieved continuous breakthroughs. Conventional PCR is widely used for the preliminary screening of various pathogens (1, 20). Comparative studies of different molecular techniques, including PCR, real-time PCR, and LAMP, have demonstrated their respective advantages in veterinary diagnostic applications (24). Quantitative real-time PCR (qPCR), owing to its high sensitivity, high specificity, and quantitative capability, has become the mainstream technology for the detection of viral and bacterial pathogens in laboratory monkeys. It is particularly demonstrating for detecting low-burden or latent pathogens such as monkeypox virus and B virus (7, 8). In recent years, diagnostic technologies based on the CRISPR/Cas gene editing system have emerged as research hotspots in the field of point-of-care testing (POCT) due to their independence from precision instruments, rapid detection speed, and relatively high sensitivity (25, 26). In the field of immunology, ELISA technology, characterized by its standardized operation and high-throughput detection capability, has become the preferred tool for antibody level monitoring and serological surveillance in monkey colonies. Immunochromatographic techniques, such as colloidal gold test strips and immunoassay combs, play an important role in on-site rapid screening owing to their simplicity and lack of equipment requirements (27). Imaging diagnostic techniques, particularly digital radiography (DR) and computed tomography (CT), are of significant value in the diagnosis of respiratory and skeletal system diseases in laboratory monkeys, allowing for the intuitive assessment of lesion extent and severity.

### 2.3 Research hotspots in diagnostic techniques

With technological advancements, research hotspots in the diagnostic field are converging on two major directions. The first is POCT (28, 29), which aims to simplify complex laboratory testing procedures and miniaturize testing equipment. It also aims to develop portable devices that can be used in monkey breeding facilities, quarantine stations, or even field settings. Microfluidic chips, biosensors, and portable nucleic acid amplification instruments are the main research and development directions for POCT. The second is multiplex pathogen detection (18, 19), which screens for multiple pathogens simultaneously in a single test. Laboratory monkeys are frequently co-infected with various pathogens, and multiplex detection not only significantly improves diagnostic efficiency and saves samples and costs, but also provides comprehensive etiological information for complex co-infection cases. Multiplex detection platforms based on multiplex qPCR, liquid chip technology, and high-throughput sequencing represent the current research frontiers.

## 3 Application progress of mainstream veterinary diagnostic technologies

When interpreting the performance metrics reported in the following sections, readers should bear in mind that diagnostic sensitivity and specificity are not inherent properties of a technology, but depend on the specific assay format, pathogen, specimen type, target population, reference standard, and study design. All numerical values cited are derived from specific studies and should not be generalized as universal performance characteristics of the diagnostic platform.

### 3.1 Molecular diagnostic technologies

#### 3.1.1 Conventional PCR

Conventional PCR detects amplification products by gel electrophoresis to determine results (30). In the diagnosis of infectious diseases in laboratory monkeys, it is primarily used for qualitative detection, suitable for samples with high pathogen loads or for preliminary pathogen screening. Han et al. (31) established a Touchdown PCR method for direct detection of TB DNA from whole blood of monkeys by amplifying the conserved region ESAT-6 specific to TB, demonstrating high sensitivity and specificity as well as simplicity. Wang et al. (32) used primer sequences targeting the 16S rRNA of Helicobacter genus, 16S rRNA of *Helicobacter pylori*, and newly designed 16S rRNA-specific primers for “*Macaca mulatta Helicobacter*” to detect Helicobacter infections in fecal samples of rhesus monkeys and cynomolgus monkeys. The infection rates of *Helicobacter pylori* and “*Macaca mulatta Helicobacter*” were both 100% in rhesus monkeys, while in cynomolgus monkeys, the infection rates were 100 and 97.8%, respectively. Zhang et al. (22) also used PCR technology for rapid identification of suspected *Mycobacterium* strains isolated from monkeys, greatly shortening the identification period. Although conventional PCR is classic and reliable, its multiple operational steps, long turnaround time, susceptibility to cross-contamination leading to false positives, and inability to perform quantitative analysis limit its application scenarios.

#### 3.1.2 Quantitative real-time PCR (qPCR)

qPCR enables real-time monitoring of the amplification process by adding fluorescent dyes or fluorescent probes into the PCR reaction system and utilizing the accumulation of fluorescent signals, thereby achieving a transition from qualitative to precise quantitative analysis (33). Studies have shown that the established qPCR can detect B virus DNA in 50 samples with 100% sensitivity, and the assay is highly reproducible, with intra-assay and inter-assay coefficients of variation of 0.6 and 2.4%, respectively (34). Sun and Zhao (35) designed specific primers and probes targeting the F3L gene of monkeypox virus and established a qPCR detection method for monkeypox virus, achieving a limit of detection (LOD) of 5.1 copies/μl for the recombinant plasmid standard and demonstrating strong sensitivity, specificity, and stability. Similarly, Wang et al. (36) established a qPCR method for detecting SIV, with a detection sensitivity of 200 copies/μl, high specificity and sensitivity, and good stability. The method can be used for quantitative determination of SIV nucleic acid copy numbers. These studies confirm that the qPCR assay enables early detection of pathogens at low viral loads, a key advantage for preventing the transmission of infectious diseases in monkeys. Beyond NHP applications, qPCR has been widely employed in other veterinary species for detecting bacterial and viral pathogens, including Mycoplasma bovis (37), duck hepatitis virus (38), and methicillin-resistant Staphylococcus aureus (39). Advanced sample preparation techniques, such as immunomagnetic bead-based enrichment combined with qPCR, have also been developed for rapid pathogen detection (40).

#### 3.1.3 Digital PCR (dPCR)

The principle of dPCR involves dispersing pathogen DNA samples into tens of thousands of microdroplets, each containing a single target molecule. By statistically analyzing the results from each droplet, precise quantification of pathogen DNA is achieved (41, 42). Yang et al. (43) developed a highly sensitive dPCR assay for monkeypox virus, with an LOD of 45 copies/ml. For the diagnosis of infectious diseases in laboratory monkeys, this method has the potential for detecting low-abundance pathogens, precisely monitoring minor differences in viral load, and identifying drug resistance mutations. In their study on ultrasensitive detection and quantification of viral nucleic acids via Raindance droplet digital PCR (ddPCR), Long et al. (44) used cells and tissues from SIV-infected rhesus macaques as models to recover and quantify viral DNA and RNA. They then adopted ultrasensitive ddPCR and reverse transcription ddPCR (RT-ddPCR) on the Raindance platform for the respective quantification of viral DNA and RNA. This approach can be easily applied to numerous scenarios that demand high - sensitivity nucleic acid analysis.

Beyond viral pathogens, dPCR has demonstrated significant utility in detecting parasitic and bacterial infections in NHPs. For instance, dPCR exhibits superior diagnostic performance for *Toxoplasma gondii* detection compared to qPCR, with a limit of detection as low as 0.1 parasite/μl (45), and has been successfully applied to screen captive squirrel monkeys (12). Similarly, dPCR has been employed for the absolute quantification of *Plasmodium spp*. in primate malaria models, enabling accurate monitoring of low-density parasitemia (46). In tuberculosis research, Willemse et al. compared qPCR and dPCR for quantifying TB (Mtb) loads in macaque tissues, finding that dPCR detected the highest bacterial burden and showed significant correlation with CFU counts. This makes it a promising tool for vaccine efficacy evaluation and latent infection monitoring (47). For retroviral studies, Kuramitsu et al. developed a novel multiplex dPCR method for detecting HTLV-1 proviral deletions and applied it to the highly homologous STLV-1 genome in Japanese macaques (48). These applications confirm that dPCR is not limited to low - abundance viral detection but also provides precise quantification for a broad spectrum of pathogens in laboratory monkeys.

#### 3.1.4 Gene sequencing technology

Gene sequencing technology provides complete genomic information of pathogens, with primary applications including pathogen identification and source tracing, mutation analysis, and drug resistance monitoring (49). Zhao et al. (50) combined high-throughput evolutionary genomic analysis with whole-genome DNA methylation analysis, using Illumina and Nanopore sequencing platforms, to comprehensively characterize two clinically extracted, locally collected Monkeypox virus (MPXV) isolates. The results showed that both isolates belonged to the ancestral C.1.1 lineage and diverged into distinct branches (E.3 and E.4), indicating at least two independent viral introductions into the region, both with limited local transmission. Huang et al. (51) compared amplicon-based and metagenomic strategies for monkeypox virus whole-genome sequencing and concluded that both amplicon sequencing and metagenomic sequencing can be flexibly applied to monkeypox virus whole-genome sequencing. Specifically, amplicon sequencing yields a greater amount of effective data, whereas metagenomic sequencing demonstrates better uniformity and accuracy in sequence coverage. Wu et al. (52) conducted whole-genome sequencing and phylogenetic analysis of four monkeypox cases in Guizhou Province from 2023 to 2024. Samples from the four cases were collected, and amplicon sequencing was used to obtain complete viral genome sequences. The results indicated that monkeypox virus exhibits mutation and diversity in China, with its evolutionary dynamics showing a clear time-dependent pattern. Continuous viral genomic surveillance and close monitoring of virus origins, transmission chains, and mutations are strongly recommended.

#### 3.1.5 CRISPR/Cas-based nucleic acid detection technology

In recent years, nucleic acid detection technologies based on the CRISPR/Cas system have emerged as one of the most promising technologies in the field of molecular diagnostics. This technology utilizes Cas proteins that, upon specific recognition of the target nucleic acid, activate their non-specific trans-cleavage activity, thereby cleaving abundant fluorescent reporter molecules present in the system and generating detectable signals (53). Zhang et al. (26) developed an ultrasensitive and specific CRISPR/Cas13a-mediated POC detection method and performed clinical testing on 202 monkeypox samples and 104 interfering samples. The results showed 100% sensitivity and 100% specificity. Compared with qPCR, this method detected three positive samples, indicating its superior performance. When the method is combined with a lateral flow strip, its accessibility and simplicity provide an alternative POC diagnostic approach for MPXV detection in remote and resource-limited areas. Lu (54) established a rapid diagnostic system for monkeypox virus based on the CRISPR/Cas12j system. To enable visual detection for convenient use, colloidal gold lateral flow chromatography was incorporated into the developed detection system, achieving a sensitivity of 10^3^ copies/μl. To further improve detection sensitivity, the system was combined with recombinase polymerase amplification (RPA), an isothermal rapid amplification technique, increasing the sensitivity to 10^0^ copies/μl. The above studies significantly reduce the requirements for operator expertise and laboratory facilities, demonstrating great application potential for rapid on-site screening in resource-limited settings.

Recent studies have further expanded the application of CRISPR-based diagnostics in NHP disease detection. Huang et al. developed a thermally programmed one-pot CRISPR assay (TRACE) that meets ASSURED standards for POCT, achieving performance comparable to qPCR while reducing turnaround time 5-fold in outpatient settings (25). This advancement is particularly relevant for field deployment in resource-limited primate facilities where sophisticated laboratory infrastructure is unavailable. The combination of CRISPR/Cas systems with isothermal amplification techniques such as RPA and LAMP has significantly lowered the detection limit to as few as 100 copies/μl of viral genome, as demonstrated in monkeypox virus detection systems (25, 54). These developments substantially reduce requirements for operator expertise and laboratory facilities, demonstrating great application potential for rapid on-site screening in endemic regions and quarantine stations. Despite these encouraging advances, several practical challenges remain. First, assay standardization and guide RNA design lack unified criteria, and most platforms rely on pre-amplification steps (e.g., RPA, LAMP), which introduce contamination risks and potential false positives. Second, off-target effects and the collateral cleavage activity of Cas13a/Cas12a may generate non-specific signals, and reproducibility across laboratories requires further validation through large-scale multicenter studies. Third, reagent stability, equipment requirements, and regulatory hurdles currently limit widespread clinical deployment. Importantly, the 100% sensitivity and specificity reported in some validation studies should not be interpreted as inherent properties of CRISPR diagnostics, but rather as performance metrics achieved under optimized conditions with specific targets and sample types. Accordingly, while CRISPR/Cas holds great promise, further analytical and clinical validation is required before it can be adopted as a routine diagnostic tool for laboratory monkeys.

### 3.2 Immunological diagnostic techniques

#### 3.2.1 Enzyme-linked immunosorbent assay (ELISA)

ELISA is currently the most widely used immunological diagnostic technique. By coating antigens or antibodies onto 96-well plates and utilizing enzyme-labeled antibodies and chromogenic substrates for reaction, qualitative or quantitative detection is achieved based on color changes (55). ELISA is mature, stable, and readily amenable to automation and high-throughput operation, making it the preferred method for antibody screening in health monitoring of laboratory monkey populations.

In routine surveillance programs for laboratory monkeys, periodic ELISA detection of specific antibody levels in monkey serum can effectively assess immune status and identify potentially infected individuals. Xing et al. (56) used two methods, bridging ELISA and affinity capture elution ELISA (ACE ELISA), to detect anti - drug antibody (ADA) levels against anti - tetanus toxin monoclonal antibodies in rhesus monkeys and compared the results. The sensitivity of both the bridging ELISA and the ACE ELISA was 500 ng/ml. Yang et al. (57) developed a sandwich ELISA using two recombinant IL-8 specific antibodies and a surrogate matrix. This method involves sequential incubation steps of samples, biotinylated antibodies, and horseradish peroxidase-labeled streptavidin (SA-HRP). The method was validated according to regulatory guidelines, achieving a lower limit of quantification (LLOQ) of 4.69 pg/ml. It demonstrated good accuracy and precision, showed no interference from serum matrix effects or hemolysis, and exhibited no cross-reactivity with other cytokines.

Despite its widespread use, ELISA has inherent limitations that must be acknowledged for proper result interpretation. The hook effect—caused by an excess of target antibodies that saturate both capture and detection antibodies, leading to a paradoxical decrease in signal—can produce false-negative results in samples with very high antibody titers (58). Yang et al. evaluated this effect in a bridging ELISA system for anti-drug antibody (ADA) detection in cynomolgus monkeys and confirmed the need for sample dilution protocols to avoid this pitfall (57). Another major challenge is matrix interference from serum components, which can cause non-specific binding and elevate background signals. Additionally, false-positive reactions are common in Old World primates due to natural antibodies cross-reacting with contaminants in viral antigen preparations. A comparative study by Li et al. showed that different ELISA formats yielded varying specificity rates for B virus antibody detection in macaque sera (23). To mitigate this, the use of recombinant antigens rather than whole-virus lysates and the adoption of confirmatory testing (e.g., Western blot or competitive ELISA) are strongly recommended for routine health monitoring (23, 59) (Figure 1) (60).

**Figure 1:**
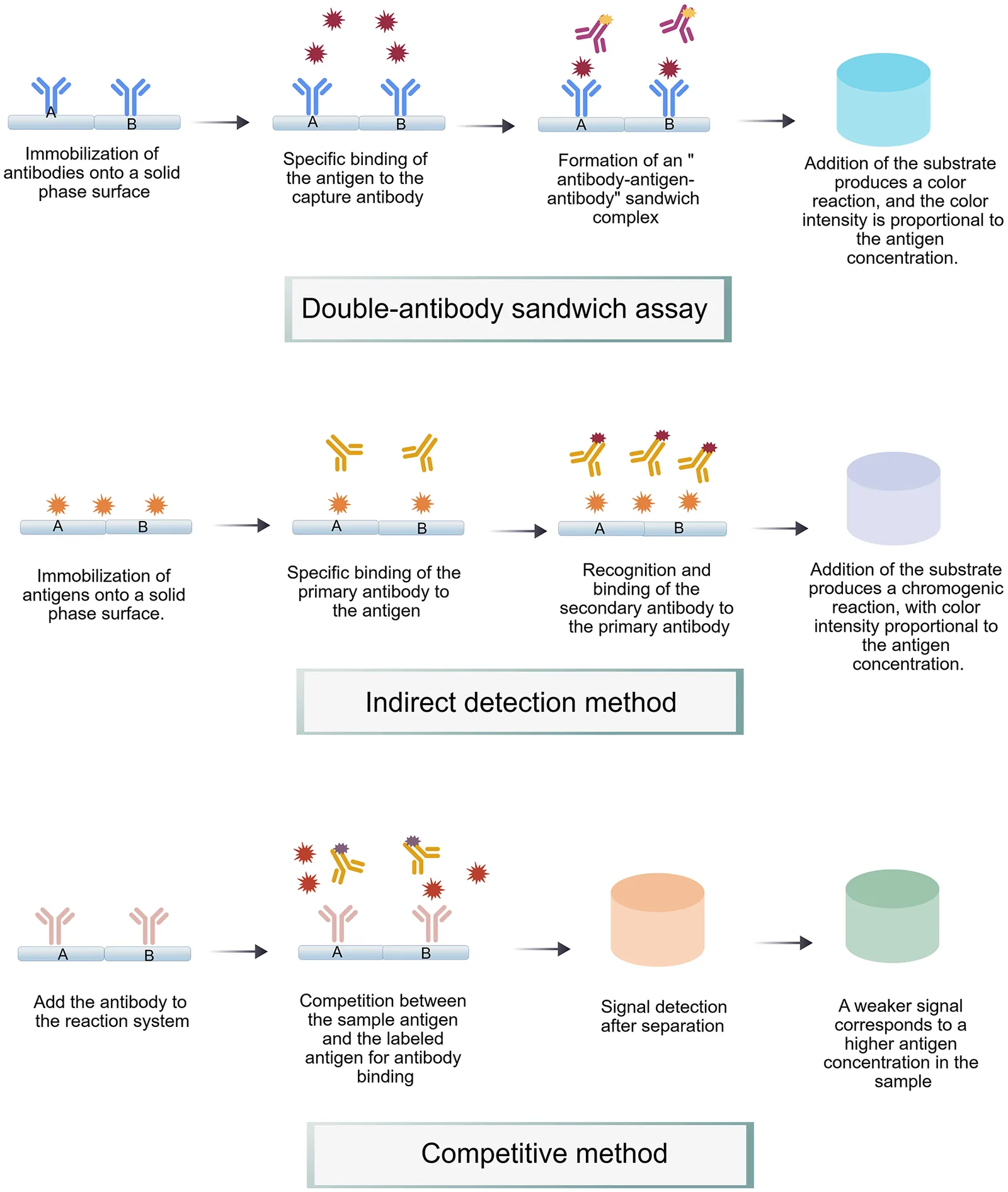
Principle of the ELISA reaction.

Interpretation of serological results in context. Serological test interpretation must consider infection stage and antibody kinetics. During the acute phase, detectable antibodies may be absent due to the window period, and false-negative results can occur if samples are collected too early. For definitive serological confirmation, paired sera (acute and convalescent) demonstrating a four-fold rise in antibody titer are often required. In some cases, neutralization assays may be necessary to confirm specificity, particularly when cross-reactivity among related pathogens is a concern. Antigen detection assays, which directly detect pathogen components, can complement serology by confirming active infection in the early stages before antibody responses are detectable. The combined interpretation of molecular and serological results is strongly recommended: molecular tests indicate current infection, while serology reflects past exposure or immune response. Together, they provide a more complete diagnostic picture and help distinguish active from resolved or latent infections.

#### 3.2.2 Immunofluorescence techniques

Immunofluorescence assay (IFA) and immunohistochemistry (IHC) are primarily used for the localization and detection of pathogens at the cellular or tissue level (61, 62). The principle involves the specific binding of fluorochrome- or enzyme-labeled antibodies to pathogen antigens in cells or tissue sections. This allows direct visualization of the distribution of pathogens within tissues and the types of infected cells under a fluorescence microscope or conventional light microscope. He et al. (63) established a multiplex immunofluorescence staining method combined with tyramide signal amplification (TSA), as well as the corresponding quantitative analysis protocol. This technique provides a powerful tool for in-depth pathological research and aids investigations into the pathogenesis of simian Acquired immune deficiency syndrome (AIDS) and other diseases in macaques. Mariotti et al. (64) used indirect immunofluorescence for the diagnosis of bullous pemphigoid and mucous membrane pemphigoid, incubating cryostat sections with serum samples in BSA-PBS buffer, and observing bound IgG using fluorescein isothiocyanate (FITC)-conjugated secondary antibodies. Liu et al. (65) constructed a plasmid expressing p27 protein of simian type D retrovirus and established a flow immunofluorescence microsphere detection technology for simian type D retrovirus. They successfully expressed the p27 protein and developed a flow immunofluorescence microsphere assay characterized by high sensitivity, strong specificity, and low sample requirements.

While IFA and IHC are powerful for pathogen localization, their major drawbacks include subjectivity in result interpretation, dependence on high-quality fluorophores, and the need for specialized equipment and skilled pathologists. Studies on imaging evaluation have demonstrated significant observer variations in interpreting similar diagnostic signals (66, 67). Moreover, the technique is not amenable to high-throughput screening. To overcome these, TSA has been integrated into multiplex IFA protocols, substantially enhancing signal intensity and enabling simultaneous detection of multiple antigens within the same tissue section, as demonstrated in simian AIDS pathology studies (63). Automation of image acquisition and AI-assisted quantification are emerging as promising solutions to reduce observer variability (68).

#### 3.2.3 Colloidal gold immunochromatographic assay (GICA)

Colloidal Gold Immunochromatographic Assay is a typical POCT technology. Its principle relies on capillary action, allowing the sample liquid to migrate along a nitrocellulose membrane and react with antigens or antibodies immobilized on specific regions of the membrane. Results are determined by color development of the colloidal gold conjugate at the test line (T line) (69). The most significant advantages of GICA are its rapidity, simplicity, and independence from any instrumentation. Results are typically available within 10–15 min, making it highly suitable for large-scale rapid screening and preliminary diagnosis at monkey breeding facilities, animal quarantine ports, and other field settings.

The immunocomp assay for SIV antibodies demonstrates high sensitivity, strong specificity, and good reproducibility. Compared with ELISA kits, the coincidence rate reaches 100%, indicating excellent accuracy and application value (70). After testing 10 suspected monkey serum samples, the immunocomp assay for simian measles virus antibodies showed a consistency rate of 100.0% with ELISA results (Kappa = 1.000), making it suitable for rapid assessment of measles immunization status in monkey colonies and quarantine of wild-sourced monkeys (11).

Although GICA technology offers numerous advantages, its limitations are also evident. Compared with molecular biology methods and ELISA, its sensitivity is generally lower (71, 72), which may lead to false-negative results during early infection or when antibody levels are low. Therefore, GICA is typically used as a preliminary screening tool.

Despite its operational simplicity, GICA suffers from relatively low sensitivity compared to ELISA and molecular methods. Comparative studies have shown that lateral-flow devices generally exhibit lower diagnostic sensitivity than qPCR and ELISA, particularly during early infection stages or when antibody titers are low (71, 72). White et al. demonstrated that the sensitivity of lateral-flow devices for invasive aspergillosis diagnosis was significantly inferior to that of galactomannan ELISA and real-time PCR (72). The primary reasons include the limited antibody loading capacity on nitrocellulose membranes, the weak signal output of colloidal gold, and the lack of an amplification step (69). False-negative results are particularly problematic for detecting low-level antibodies in early infection (73). To improve sensitivity, several strategies have been explored: (i) nanomaterial-enhanced labels (69); (ii) signal amplification systems combining enzymatic reactions with gold conjugates (68); and (iii) pre-concentration steps to enrich target antibodies from larger sample volumes. Future POC devices may integrate GICA with isothermal nucleic acid amplification for simultaneous antigen and nucleic acid detection (54, 74). Currently, GICA is most suitable for rapid preliminary screening in quarantine stations and breeding facilities, but positive results should always be confirmed by ELISA or qPCR before making definitive diagnostic or management decisions (27, 73).

### 3.3 Imaging diagnostic techniques

#### 3.3.1 Ultrasound

Ultrasound diagnostics utilizes the reflection characteristics of ultrasonic waves at different tissue interfaces to generate images. It offers advantages such as real-time imaging, absence of radiation, flexible operation, and repeatability (75). Xing et al. (76) evaluated the feasibility of 3D ultrasound localization microscopy (ULM) for the brain in NHPs using a 256-channel programmable ultrasound scanner. Their study demonstrated that using an 8 MHz multiplexed matrix probe following craniotomy and durectomy, high-resolution vascular maps of the macaque brain could be obtained at large depths (up to 3 cm). The technique could distinguish vessels as small as 26.9 μm. They also demonstrated that transcranial imaging of the macaque brain at similar depths is feasible using a 3 MHz probe, achieving a resolution of 60 μm. Ultrasound is also used for pregnancy monitoring, fetal development assessment, and follicular development monitoring in laboratory monkeys, playing a key role in assisted reproduction research and breeding management. The diagnostic accuracy for macaque pregnancy is 95.5% at 15 days of gestation and 100% at 20 days of gestation (77). However, the accuracy of ultrasound diagnostics is highly dependent on operator experience and skill level. Moreover, its penetration is limited in air-containing or dense tissues such as the lungs and bones, resulting in suboptimal imaging quality.

#### 3.3.2 Digital radiography (DR) and computed tomography (CT)

DR and CT are both X-ray-based imaging techniques that can clearly display bone structures as well as the contours and density changes of thoracic and abdominal organs (78, 79). Beyond conventional radiography and CT, advanced imaging modalities such as MRI have also been explored for translational applications in both human and veterinary oncology, providing valuable insights for NHP models (80). One study indicated that based on imaging and pathological findings, a rhesus monkey was diagnosed with esophageal squamous cell carcinoma (ESCC) and underwent surgical treatment. This provides an important reference for the diagnosis and treatment of ESCC in rhesus monkeys and also provides a valuable spontaneous animal model for future ESCC research (81). Munster et al. (82) demonstrated that SARS-CoV-2 causes respiratory disease in rhesus monkeys lasting between 8 and 16 days. Pulmonary infiltration, a hallmark feature of COVID-19 in humans, was visualized on chest X-rays, establishing the rhesus monkey as a model for SARS-CoV-2 infection. Lin et al. (83) demonstrated that serial PET-CT using an FDG probe can track overall disease, individual granulomas, and lymph nodes during TB infection and treatment in an animal disease model that closely resembles human disease. Using PET-CT, the efficacy of single-agent or multi-agent regimens can be determined within 1–2 months after treatment initiation.

In the context of infectious diseases, imaging techniques, particularly ultrasound, DR, and CT, play several important roles. First, they enable detection of characteristic lesions, such as pulmonary infiltrates, granulomas, and skeletal abnormalities. Second, they facilitate disease severity assessment by quantifying lesion extent, distribution, and progression over time. Third, imaging findings contribute to differential diagnosis by helping distinguish infectious from neoplastic or non-infectious inflammatory conditions. Fourth, serial imaging allows monitoring of treatment response, particularly in TB cases. Finally, imaging provides valuable pathological correlation when combined with biopsy or necropsy findings, enhancing diagnostic accuracy and understanding of disease pathogenesis (84). However, practical factors must also be considered: anesthesia or sedation is typically required for image acquisition in NHPs to prevent motion artifacts; radiation exposure from DR and CT necessitates dose optimization, especially for longitudinal studies; equipment availability may be limited in some facilities; animal handling requires trained personnel; and operator expertise significantly influences image quality and interpretation, particularly for ultrasound (82). These factors should be weighed when incorporating imaging into the diagnostic workup of infectious diseases in laboratory monkeys.

### 3.4 Traditional microbiological diagnostic techniques

#### 3.4.1 Pathogen isolation and culture

Pathogen isolation and culture represent the definitive reference for diagnosing bacterial and certain viral diseases. Comparative evaluations of different identification methods, including PCR, qPCR, and culture, have demonstrated the complementary roles of these approaches in bacterial pathogen detection (85). In virology, cell culture remains an essential tool for virus isolation and characterization, despite challenges in establishing and maintaining appropriate culture systems (86). Successfully isolating and identifying a pathogen from sterile sites or lesional tissues constitutes the most direct and reliable evidence for etiological diagnosis. Li et al. (87) isolated and identified a strain (RM2023) from the secretions of a rhesus monkey with endometritis as *Morganella morganii*. Morphological results showed that the strain exhibited round, convex, grayish-white colonies with a smooth surface and a diameter ranging from 1 to 2 mm when cultured on Columbia blood agar at 37 °C for 24 h. On Salmonella-Shigella (S.S.) agar at 37 °C for 24 h, the colonies appeared round, flat, and translucent. Gram staining revealed negative results, with blunt ends and no spore-forming characteristics. Molecular biological results indicated that the 16S rRNA sequence of this strain showed more than 96.9% similarity to published *Morganella morganii* sequences from various sources in the NCBI GenBank database. Similarly, in the diagnosis of infectious dysentery in Yunnan snub-nosed monkeys and streptococcal lobar pneumonia in macaques, pathogen isolation and culture are also key steps for confirmation (88, 89). The value of pathogen isolation lies not only in confirmation but also, more importantly, in obtaining live bacterial or viral strains, which serve as the foundation for subsequent research. However, pathogen isolation has drawbacks, including being time-consuming, operationally complex, requiring high-level laboratory conditions, and being unsuitable for many viruses that cannot be routinely cultured (22).

It should be noted, however, that the choice of reference standard is pathogen-dependent. While culture serves as the definitive reference for cultivable bacterial pathogens such as TB, molecular methods (e.g., qPCR) or serological assays are more appropriate for viral infections. Composite reference standards combining multiple modalities may be required for difficult-to-culture organisms.

#### 3.4.2 Antimicrobial susceptibility testing (AST)

Once a pathogenic bacterium is isolated in the diagnosis of bacterial diseases, performing antimicrobial susceptibility testing (AST) is critically important. AST determines the susceptibility of the bacterial strain to various antimicrobial agents, providing direct scientific evidence for clinical veterinarians to select effective and appropriate drugs. This not only improves treatment success rates but also effectively avoids treatment failure and antibiotic misuse that may result from empirical medication, thereby delaying the emergence of drug resistance. Multiple reports on the diagnosis and treatment of bacterial diseases in laboratory monkeys have emphasized the importance of guiding medication based on AST results (90–92). In the study by Li et al. (87) on the isolation, identification, and antimicrobial susceptibility of *Morganella morganii* in Chinese rhesus monkeys (*Macaca mulatta*), the susceptibility testing results showed that the isolate (RM2023) was sensitive to ceftriaxone, gentamicin, and tetracycline; intermediately sensitive to ampicillin; and resistant to penicillin, vancomycin, and other antibiotics. These findings provide valuable insights for disease prevention in rhesus monkeys and contribute to molecular epidemiological research.

To complement the technology-focused structure of this review and provide a more clinically oriented reference, this table summarizes recommended diagnostic approaches for the major pathogens discussed in this paper. For each pathogen, the table presents its preferred specimen types, screening tests, confirmatory tests, complementary tests, and references. This pathogen-centered summary is designed to help veterinarians and researchers select appropriate diagnostic workflows based on the suspected infectious agent (Table 1). The evolution of veterinary diagnostic techniques for laboratory monkeys, from traditional microbial culture to molecular diagnostics, is illustrated in Figure 2.

**Table 1:** Pathogen-oriented diagnostic recommendations for major infectious diseases in laboratory monkeys.

Pathogen	Preferred specimen	Screening test	Confirmatory test	Complementary test	Reference(s)
B virus	Oral swab, blood, CSF	ELISA, immunocomb	qPCR (viral DNA)	Western blot	(23, 27, 34)
TB	BALF, gastric lavage, tissue	TST, IGRA	qPCR (IS6110/ESAT-6), culture	Chest DR/CT, PET-CT	(22, 31, 83)
MPXV	Skin lesion swab, blood	qPCR (F3L gene)	qPCR, WGS	CRISPR-based POC	(25, 26, 43)
SIV	Blood, plasma	ELISA (antibodies)	qPCR (viral RNA), Western blot	Immunocomb assay	(36, 57, 70)
Toxoplasma gondii	Blood, CSF, tissue	ELISA (serology)	qPCR, dPCR	IFA/IHC	(12, 45)

TST, tuberculin skin test; IGRA, interferon-gamma release assay; WGS, whole-genome sequencing; POC, point-of-care; CSF, cerebrospinal fluid; BALF, bronchoalveolar lavage fluid.

**Figure 2:**
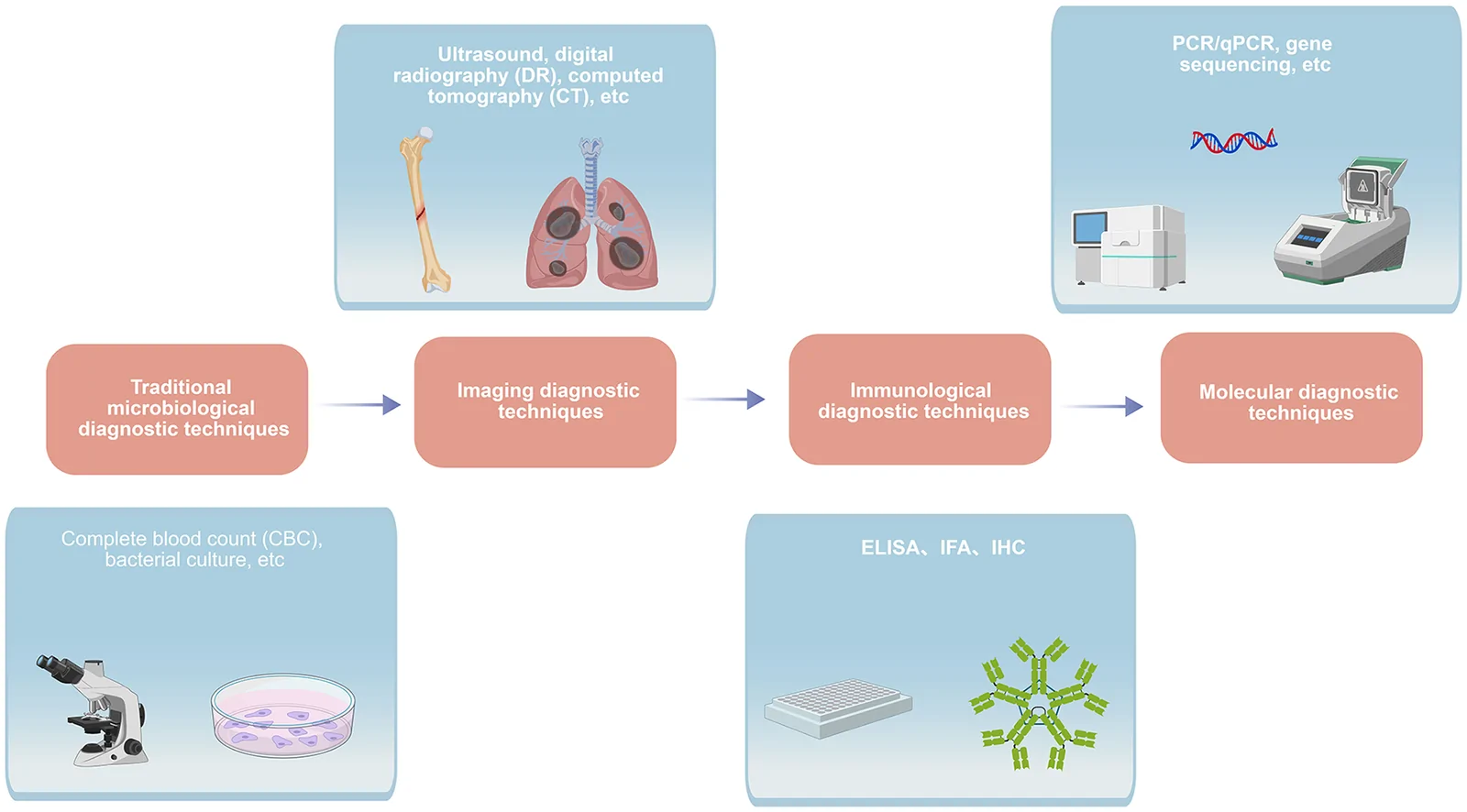
Evolution of veterinary diagnostic techniques.

## 4 Comparison and applicable scenarios of different diagnostic techniques

Sample selection and timing are critical for diagnostic accuracy. The applicability of each technique varies with specimen type. Blood, serum, plasma, respiratory swabs, BALF, feces, urine, tissue, and lymph nodes each have specific diagnostic value and limitations for different pathogens and detection platforms. Sampling outside the optimal diagnostic window may compromise test performance. Even the most sensitive assay will not be accurate if the wrong specimen is obtained, and negative results from suboptimal samples should be interpreted with caution. Therefore, the diagnostic workflow should integrate sampling strategy with test selection based on the specific clinical scenario and target pathogen.

To more intuitively demonstrate the characteristics and applicable scopes of various diagnostic techniques, the following table provides a comparison of mainstream technologies across multiple dimensions (Table 2).

**Table 2:** Comparison of different diagnostic techniques.

Techniques	Sensitivity	Specificity	Complexity	Cost	Applications	Limitations	Reference(s)
qPCR	High (for B virus)	High	Moderate	Moderate	Laboratory precise detection, batch monitoring, low-burden pathogen detection	Requires thermal cycler; sensitive to inhibitors	(34, 93)
dPCR	High (LOD: 45 copies/mL for MPXV)	High	Moderate	High	Absolute quantification; latent infection; rare mutation detection	Higher cost; lower throughput than qPCR	(43)
CRISPR/Cas	High (study-dependent)	High (study-dependent)	Moderate (with RPA)	Moderate	Rapid on-site detection, targeted pathogen detection	Pre-amplification needed; potential carryover contamination	(26, 53)
ELISA	High (assay-dependent)	High (assay-dependent)	Moderate	Moderate	Batch antibody screening; sero-surveillance; immune status monitoring	Hook effect; matrix interference; false-positives in Old World monkeys	(108, 109)
GICA	Moderate-High (study-dependent)	Moderate	Low	Low	On-site rapid screening; emergency diagnosis; field quarantine	Lower sensitivity than ELISA/PCR; subjective readout; no quantification	(73)
Ultrasound	Moderate (operator-dependent)	Moderate-High	Moderate	Moderate	Pregnancy/fetal monitoring; abdominal/liver disease assessment	Highly operator-dependent; poor for air/bone interfaces	(110)
DR/CT	High	Moderate	High	High	Pulmonary infection (TB); skeletal injury; tumor staging	High cost; radiation exposure; requires anesthesia	(81, 84)
Pathogen isolation and culture	Moderate (for viable bacteria)	High	High	Moderate	Definitive standard for bacterial confirmation (pathogen-specific); drug susceptibility testing (AST)	Time-consuming (weeks); requires BSL-3 for TB; not for all pathogens	(22, 87)
IFA/IHC	High (study-dependent)	High	Moderate	Moderate	Pathogen localization; pathological diagnosis; confirmatory testing	Subjective interpretation; requires specialized equipment and expertise	(65)

Each diagnostic technique possesses its own advantageous characteristics and application limitations, and at present, there is no ideal diagnostic method applicable to all testing scenarios. In the practice of infectious disease prevention and control in laboratory monkeys, a scientific and rational selection of single or combined technical solutions should be made based on the diagnostic objectives, cost budget, timeliness requirements, and available resource conditions. This establishes a multi-level, three-dimensional diagnostic system that ranges from rapid initial screening to laboratory-based precise confirmation, and ultimately to pathological and etiological final determination.

### 4.1 Establishment of diagnostic algorithms for routine practice

Translating technical capabilities into effective clinical decisions requires the development of practical diagnostic algorithms—decision flowcharts that guide veterinarians through a logical sequence of tests based on clinical presentation, colony health history, and available infrastructure. Such algorithms optimize resource use, reduce diagnostic delays, and ensure consistent interpretation (92). For example, a tiered algorithm for suspected tuberculosis in a macaque could be structured as follows:

#### 4.1.1 Tier 1

Use GICA or interferon-gamma release assay (IGRA) on whole blood for rapid on-site screening in quarantine or during routine health checks. If negative but high clinical suspicion persists, proceed to Tier 2.

#### 4.1.2 Tier 2

Collect gastric lavage, bronchoalveolar lavage fluid (BALF), or tissue biopsies. Perform qPCR targeting TB complex-specific genes for rapid and sensitive detection (31, 34). Concurrently, run ELISA for anti-TB antibody detection.

#### 4.1.3 Tier 3

For tuberculosis and other suspected bacterial infections where culture is feasible, perform pathogen isolation and culture followed by AST. This serves as the definitive reference for these specific pathogens and guides targeted therapy (22, 87, 89).

#### 4.1.4 Tier 4

In outbreak scenarios or for source tracing, deploy whole-genome sequencing (WGS) to determine strain relatedness and drug-resistance mutations (50, 52).

Similar algorithms should be developed for other major diseases incorporating local prevalence data, cost considerations, and laboratory capability. This structured approach bridges the gap between technology availability and clinical application, enhancing both animal welfare and research data integrity (92, 93).

## 5 Technical application bottlenecks and countermeasures

### 5.1 Core bottlenecks

#### 5.1.1 High equipment costs

The acquisition costs of advanced diagnostic technologies such as dPCR, CT scanners, and high-throughput sequencing platforms are prohibitively high (42, 94). Moreover, the prices of associated imported reagents and consumables are also high, representing a considerable financial burden for most primary-level laboratory monkey breeding facilities and some research units with limited budgets. This cost pressure directly impedes the widespread adoption of these advanced technologies. Consequently, many facilities still rely on traditional methods with relatively lower sensitivity and specificity for disease surveillance, which may lead to delayed detection of infectious diseases and pose potential transmission risks.

#### 5.1.2 Uneven skill levels of professional veterinarians and technicians

The effective application of modern diagnostic technologies depends not only on advanced equipment but also, more critically, on the professional skills and experience of the operators. Tasks including qPCR primer and probe design, reaction condition optimization, result interpretation (95), ultrasound scanning and image analysis (66), as well as pathological section preparation and diagnosis (67), require training and extensive practical experience. Currently, there is a relative shortage of specialized veterinarians in the field of laboratory animal medicine, particularly at primary-level institutions, where the professional proficiency of technical personnel varies considerably (96). Non-standardized operations or erroneous result interpretations can compromise the accuracy of diagnostic findings or even lead to completely incorrect conclusions, thereby misleading subsequent prevention, control, and treatment decisions.

#### 5.1.3 Lack of unified technical standards and specifications for the diagnosis of infectious diseases in laboratory monkeys

A widely recognized and implemented set of unified industry or national standards for the diagnosis of major infectious diseases in laboratory monkeys is currently lacking. Different laboratories or institutions may adopt varying diagnostic protocols, reagent kits, and interpretation criteria, resulting in poor comparability of test results across institutions and hindering effective data integration and epidemic risk assessment.

### 5.2 Strategies

To address these barriers, strategies should be tailored to the specific constraints and needs of different facility types, and directly linked to the diagnostic challenges identified in previous sections. For academic research institutions, where specialized expertise is often available but funding may be limited, open-access protocols and inter-laboratory collaborative quality control programs can help address the lack of unified standards (31, 34) at low cost, while promoting method harmonization. For commercial laboratory-animal facilities, which handle large colony sizes and high testing volumes, scalable automation (e.g., automated nucleic acid extraction, high-throughput ELISA platforms) and centralized reference laboratories for specialized assays (e.g., WGS, dPCR) can reduce per-sample costs and improve turnaround times, addressing the high equipment costs and skilled personnel shortages. For quarantine facilities, which require rapid decisions for animal import/export and outbreak containment, investment in field-deployable POC tests [e.g., GICA, portable PCR, CRISPR-based POC platforms (25, 54)] is critical. These tools enable immediate on-site screening, reducing dependence on centralized laboratories and addressing the timeliness gaps of traditional culture and detection methods. For resource-limited settings, where equipment and trained personnel are scarce, cost-effective local solutions—such as in-house reagent preparation, equipment-sharing networks, and partnerships with external reference laboratories—can help bridge the gap. Training programs adapted to local contexts and delivered through regional veterinary networks can gradually address skill shortages (93).

Finally, the development of unified diagnostic standards should not be a top-down policy exercise. Instead, professional societies and regulatory bodies should convene experts from all facility types to develop scenario-specific guidelines that specify recommended techniques, quality control requirements, and result interpretation criteria for each diagnostic context (97). This approach ensures that standards are practical and widely adoptable, directly responding to the standardization gaps. The issuance of such standards will provide a unified basis for diagnostic work across different facilities, offer benchmarks for the registration, approval, and quality evaluation of diagnostic products, and thereby comprehensively enhance the overall level and standardization of infectious disease diagnosis in laboratory monkeys in China.

## 6 Prospects of cutting-edge technologies

### 6.1 Point-of-care testing (POCT)

POCT refers to a class of detection methods that are performed at the sampling site and can yield results rapidly (74). This is of great significance for scenarios such as routine health inspections in monkey breeding facilities, quarantine of newly introduced animals, and on-site management of disease outbreaks. Future development directions include miniaturized qPCR and isothermal amplification technologies. Isothermal amplification techniques represented by RPA and LAMP do not require precision thermal cyclers; when combined with CRISPR/Cas systems, they can be developed into molecular diagnostic methods with high sensitivity, good specificity, and operational simplicity (53, 98). Another direction is novel biosensors (99). Electrochemical or optical biosensors developed using advanced materials such as nanomaterials, graphene, and quantum dots can directly convert recognition events of pathogen nucleic acids or antigens into readable electrical or optical signals, enabling label-free, ultrasensitive, and real-time dynamic detection.

### 6.2 Multiplex pathogen detection technologies

Laboratory monkeys often face co-infections or opportunistic infections with multiple pathogens. Traditional single-pathogen detection approaches are inefficient and prone to missed diagnoses. Multiplex pathogen detection technologies, which can obtain information on multiple pathogens simultaneously from a single sample, represent an important future trend in diagnostics.

Multiplex PCR: By setting multiple pairs of primers and probes in a single reaction tube, multiple pathogens can be detected simultaneously. Multiplex qPCR kits that can simultaneously detect 12 viruses or serotypes are already available (100). Future development aims to further increase multiplexing throughput to achieve “single-tube” detection of pathogens commonly associated with a particular syndrome, such as respiratory syndrome or diarrhea syndrome. Liquid chip technology: This technology uses microspheres encoded with different fluorescent signals as reaction carriers. Each microsphere is coupled with a probe targeting a specific pathogen. Through flow cytometry analysis, high-throughput detection of antigens or antibodies against dozens or even hundreds of pathogens can be achieved in a single test (101). High-throughput sequencing (102): Metagenomic sequencing provides an unbiased approach to pathogen detection, theoretically capable of identifying all microorganisms present in a sample. Metagenomic analysis has been applied to characterize microbial communities in various clinical samples, demonstrating its utility in identifying pathogens and understanding complex microbial ecosystems (103). It is particularly suitable for discovering emerging pathogens and analyzing the pathogen spectrum of complex co-infections. As sequencing costs continue to decline rapidly, its application as an advanced diagnostic tool will become increasingly widespread.

Despite these promises, several practical impediments currently limit the routine application of metagenomic sequencing in laboratory monkey diagnostics. High background of host-derived nucleic acids reduces microbial read proportion and may obscure low-abundance pathogens. Adequate sequencing depth is essential for reliable detection, particularly in clinical samples with low microbial biomass, but increases cost and turnaround time. Contamination from reagents or the environment can introduce spurious sequences, while bioinformatics analysis requires specialized expertise, robust computational infrastructure, and curated reference databases. Interpretation of low-abundance reads remains challenging, as it is often difficult to distinguish true pathogens from colonizers, contaminants, or commensals. Therefore, metagenomic sequencing should currently be viewed as a complementary method to targeted diagnostic testing (e.g., qPCR, culture), rather than a replacement. It is particularly valuable for syndrome-based differential diagnosis, investigation of cases with high clinical suspicion but negative targeted assays, and detection of unexpected or emerging pathogens. As sequencing costs decline, bioinformatics pipelines become more accessible, and standardized interpretation criteria are established, metagenomic sequencing may progressively transition toward broader routine use.

### 6.3 Artificial intelligence (AI)-assisted diagnosis

AI, particularly deep learning, is poised to transform disease diagnosis in laboratory monkey medicine. Its applications extend far beyond simple image classification and can be categorized into a structured framework as follows:

#### 6.3.1 AI-enhanced imaging and pathological analysis

Convolutional neural networks (CNNs) have been trained to automatically segment and quantify tuberculous lesions in primate chest CTs, outperforming manual assessment in tracking disease progression. Comprehensive imaging and pathological analysis of spontaneous diseases in rhesus monkeys, such as esophageal squamous cell carcinoma, have provided valuable reference data for training AI models (81). In histopathology, machine learning models have been applied to automatically detect retinal ganglion cells in African green monkey eye sections and to classify tissue pathology in Ebola-infected NHP models (104). AI-assisted endoscopy is emerging for early detection of gastric and colorectal lesions in macaques (68).

#### 6.3.2 AI for behavioral and physiological monitoring

Computer vision systems have been deployed for continuous, non-invasive monitoring of monkey behavior and vital signs. The CapuchinAI 1.0 system achieves highly accurate individual recognition and behavioral classification in wild capuchin monkeys (105). FullMAI enables longitudinal behavioral tracking in marmosets, detecting subtle changes in posture, gait, and facial expressions that correlate with early illness or pain (106). Non-contact heart rate monitoring using millimeter-wave radar has also been validated in NHP models, offering a stress-free health surveillance tool. Such AI-driven approaches enable early detection of disease onset before clinical symptoms become apparent, significantly improving animal welfare.

#### 6.3.3 AI in brain-computer interfaces (BCI) and neurological diagnostics

In NHP models of neurological disorders, deep learning decoders are used to analyze electrocorticography (ECoG) or intracortical signals not only for motor decoding but also for identifying abnormal neural patterns as diagnostic biomarkers of disease onset and progression. Advances in deep learning-based decoders for intracortical BCIs have demonstrated the feasibility of using AI to extract clinically meaningful signals from neural recordings in awake, behaving NHPs (107).

#### 6.3.4 Integrated data predictive models

Future diagnostic systems will integrate clinical, immunological, and imaging data into AI models such as random forests or gradient boosting machines to improve disease prediction and risk assessment.

Despite its potential, AI faces significant challenges before routine NHP diagnostic deployment. These include limited training datasets, species-specific anatomical variation, lack of external validation, imaging standardization issues, poor explainability, serious consequences of false positives/negatives (especially for zoonotic pathogens), and algorithmic bias from imbalanced training data. It is also important to distinguish between AI applications that have been tested in NHPs and those extrapolated from human or other veterinary imaging; the latter are discussed as future directions requiring further validation. Therefore, AI should be viewed as a promising but not yet validated tool for routine NHP diagnostics, and rigorous prospective studies are essential before clinical adoption.

## 7 Conclusion

Veterinary diagnostic techniques are essential for safeguarding the welfare of laboratory monkeys, maintaining biosafety, and ensuring the reliability of biomedical research data. Several key takeaways emerge from this review with direct practical implications.

First, no single diagnostic technique is universally applicable. The choice of diagnostic method should be guided by the specific pathogen (e.g., bacterial, viral, or parasitic), sample type (e.g., blood, BALF, or tissue), and clinical scenario (e.g., routine screening, outbreak investigation, or confirmatory diagnosis). Second, combined diagnostic strategies that integrate molecular, immunological, imaging, and traditional microbiological approaches are more reliable than any single technology, as they provide complementary evidence across multiple diagnostic dimensions. Third, context-specific factors, including cost, equipment availability, operator expertise, and biosafety requirements, must be carefully weighed when selecting diagnostic approaches for different facility types.

To properly evaluate and deploy emerging technologies in laboratory primates, three research priorities should be addressed. First, prospective validation studies with standardized protocols and well-defined reference standards are urgently needed to establish the true performance of new diagnostic platforms under real-world conditions. Second, multi-center collaboration across different NHP facilities is essential to generate robust and generalizable evidence that accounts for population diversity and institutional variability. Third, cost-effectiveness analyses should be conducted to inform practical deployment decisions, particularly for resource-limited settings where advanced technologies may be prohibitively expensive.

In conclusion, the future of laboratory monkey diagnostics lies not in replacing established methods with emerging technologies, but in their strategic integration. Guided by clinical context and supported by rigorous validation, this integrated approach will enable accurate, efficient, and sustainable disease management that benefits both animal health and biomedical research.
